# Investigating the need for scholarly communications positions in Association of Academic Health Sciences Libraries member institutions

**DOI:** 10.5195/jmla.2017.208

**Published:** 2017-04

**Authors:** Kim Mears, Sandra L. Bandy

## Abstract

**Background:**

The role of health sciences librarians has expanded in the scholarly communications landscape as a result of the increase in federal public access mandates and the continued expansion of publishing avenues. This has created the need to investigate whether academic health sciences libraries should have scholarly communications positions to provide education and services exclusively related to scholarly communication topics.

**Methods:**

A nine-question online survey was distributed through the Association of Academic Health Sciences Libraries (AAHSL) email discussion list to gather preliminary findings from and opinions of directors of health sciences libraries on the need for scholarly communications positions.

**Results:**

The survey received a 38% response rate. The authors found that AAHSL members are currently providing scholarly communications services, and 46% of respondents expressed the need to devote a full-time position to this role.

**Discussion:**

Our survey reveals a juxtaposition occurring in AAHSL member libraries. While administrators acknowledge the need to provide scholarly communications services, they often experience budget challenges in providing a full-time position for these services.

## INTRODUCTION

More than ten years have passed since the Budapest Open Access Initiative, the first open access movement, was established [1]. These widely known principles led to changes in the ways scholarly literature is disseminated, such as transitioning from traditional print to electronic distribution of works without subscription barriers. As a result of this change, libraries experienced the progression from “gatekeepers of knowledge” toward a dissemination and publication role, moving librarianship “beyond a custodial role model” and into one that involves “active...contributi[ons] to the evolution of scholarly communication” [2].

Defined as “the system through which research and other scholarly writings are created, evaluated for quality, disseminated to the scholarly community, and preserved for future use” [3], the scholarly communications landscape has exponentially increased and resulted in changes in institutional needs from libraries. Librarians are meeting these needs by providing new services. Health sciences librarians are particularly well placed to take active roles in this area because of the high rate of publication in health professions as well as the recent increase in federally mandated open access policies for research. One informal measurement of this change is the increasing number of papers and posters presented at Medical Library Association (MLA) annual meetings in the area of scholarly communications, focusing on topics such as the National Institutes of Health’s (NIH’s) public access policy [4], data management, scholarly impact with altmetrics, and publishing identities. In 2010, only seven papers and posters were identified in the area of scholarly communications, compared to over thirty in 2016.

To provide support for health sciences librarians who are becoming active in this new role, MLA introduced the Ad Hoc Committee for Advocating Scholarly Communications in 2008 to discuss scholarly communications initiatives; it became the standing Scholarly Communications Committee in 2012. This committee “provides leadership within the association about trends and issues in scholarly publication and their impact on biomedical libraries, and recommends and implements programs, special initiatives and other activities in this area” [5]. The Association of Academic Health Sciences Libraries (AAHSL) also provides support for health sciences librarians addressing scholarly communications issues through its own committee.

With the formation of these new professional association committees, has there been a similar increase in scholarly communications job positions in health sciences libraries? To answer this question, the authors investigated and gathered preliminary opinions from AAHSL members on the need for a full-time scholarly communications position.

## METHODS

A web-based survey with nine questions was distributed to members of the AAHSL Directors email discussion list through SurveyMonkey. This list comprises associate members of AAHSL, which includes health sciences libraries from the United States and Canada [6]. The survey remained open for two weeks with one reminder email to encourage participation. The survey was approved by the Institutional Review Board of Augusta University.

The survey was created by reviewing recent scholarly communications–related job descriptions posted on library job boards and professional organization websites. Questions were based on the required and preferred library skills that were specified in the employment notices and included dichotomous, multiple-choice, and open-ended answers. We asked about current scholarly communications services, the title of the librarians responsible for offering scholarly communications services, the presence of a web page dedicated to scholarly communications that the library maintained, and the library’s involvement in university initiatives such as open access mandates or policies. We also asked if AAHSL members saw a need for a full-time scholarly communications librarian based on their answers to previous questions. The survey instrument is available in the supplemental appendix.

## RESULTS

**Figure 1:**
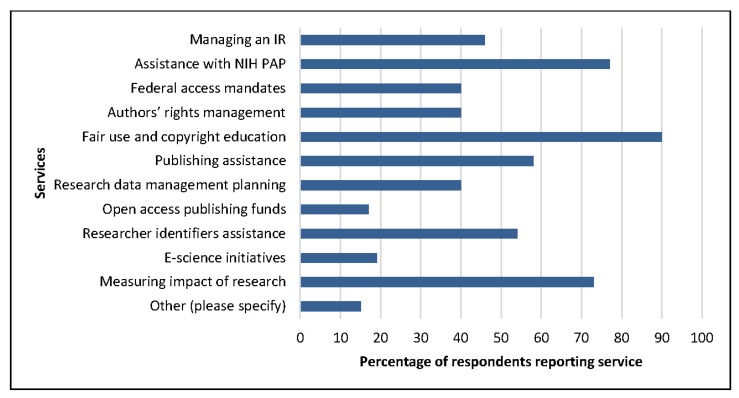
Association of Academic Health Sciences Libraries (AAHSL) members’ reported scholarly communications services IR=institutional repository; NIH PAP=National Institutes of Health public access policy.

Our survey yielded a 38% response rate (60 of 156 members). The most commonly offered services included education on fair use and copyright, assistance in complying with NIH’s public access policy, and assistance in measuring the impact of scholarly works. Over half of respondents provided education on researcher identifiers and consultations on publishing (Figure 1). Other services that were offered include serving as an NIH publisher, providing instruction on SciENcv, and serving as a writing center for manuscript or publication support. Most (61%) AAHSL members hosted a web page dedicated to scholarly communications.

In response to questions regarding participants’ engagement with open access, 27% of college or university senates had a resolution in place and 4% had a mandate. Most (59%) libraries did not participate in internationally celebrated “Open Access Week” activities.

Slightly more than half (54%) of the libraries dedicated a librarian as the primary lead in offering scholarly communications services. When asked to give the title of the librarian responsible for offering scholarly communications services, respondents reported titles such as “cataloging librarian,” “scholarly publishing librarian,” and “assistant director.” Overall, 46% of respondents thought it was necessary to devote a full-time librarian to providing scholarly communications services.

Analysis of open-ended responses found several themes, including recognizing the importance of addressing scholarly communications topics, the importance of offering scholarly communications services, and the need to think ahead in providing staffing for scholarly communications services. Multiple responses also acknowledged challenges with budget and/or staffing limitations.

## DISCUSSION

While many academic libraries have scholarly communications librarian positions, health sciences libraries are also experiencing a surge in reference and instruction needs in these areas. However, literature on scholarly communications librarian positions specifically in health sciences libraries is scarce. Cooper and Crum found that scholarly communications librarian positions emerged as a new role when they examined job announcements posted between 2008 and 2012 but did not find evidence of this trend in the literature [7]. Responsibilities of this new role found in the job descriptions included promoting the institution’s institutional repository, exploring new publishing models, assisting users while interacting with editors and/or publishers, providing support for federally mandated open access policies, and providing open access materials [7].

Our survey results indicate that AAHSL members are offering a multitude of these services, along with research data management planning, copyright consultations, and measurement of research impact. While many of these services are not unique to health sciences libraries, it is important to acknowledge that the time and manpower needed to lead scholarly communication initiatives is compounded by the prolific nature of publishing in the medical and health professions fields. The breadth and depth of services offered are an indicator that libraries are being responsive to faculty and student needs, but our survey results also reveal that AAHSL members are not engaging in the broader scholarly communications community through global initiatives like “Open Access Week.” Perhaps this is the result of not tasking a position as a leader in this area.

A challenge that AAHSL members continued to express in open-ended responses was the desire to have a scholarly communications position, but staffing and/or budget limitations often prevented creating a new position or reorganizing current staffing to offer these services. A survey of Association of Research Libraries showed that, in the majority of libraries, a single person served as the main leader of scholarly communications initiatives, with this librarian often holding the title of department head, assistant director, or scholarly communications librarian [8]. Our survey indicated the same trend of a single librarian being tasked with leading scholarly communications services and information, but open-ended questions suggested that AAHSL member libraries also used other models. Some respondents specified that a team of librarians shared responsibilities or that scholarly communications services were offered through the main academic library to the entire university. The literature confirms that these are common approaches that libraries use when offering scholarly communications services without adding new employees. The University of Minnesota Libraries [9] and the University of British Columbia Library [10] are examples of libraries incorporating scholarly communications duties into reference and instruction roles. Interestingly, the University of British Columbia Library consulted with medical and health sciences librarians specifically to “help define the library’s strategy to support our faculty and researchers who receive grants with new public access mandates” [10].

The question that remains after reviewing the results of our survey is how AAHSL members intend to provide expertise in current scholarly communications issues that students and faculty face without additional funding for a new position. Adding duties to reference and instruction librarian responsibilities is often seen as the solution, but this may not be viable in the long term because the scholarly communications landscape has significantly expanded. Job satisfaction, burnout, and employee turnover can be affected. AAHSL members must be creative in integrating a scholarly communications position into their organizational structures. As this study is only preliminary, further research is needed to investigate full-time scholarly communications positions in academic health sciences libraries.

Limitations of this study included the use of a small convenience sample and the use of a survey to collect data. The survey sample represented only a portion of academic health sciences libraries throughout the United States and Canada, so the results might not be generalizable to all health sciences libraries. Challenges that emerged from the use of a survey included that respondents did not answer all questions and that the information is self-reported. Furthermore, it is possible that the wording of questions may have led respondents to answer the survey with the entire university library system in mind instead of only the health sciences library.

## Supplemental File

Appendix Survey

## References

[1] Chan L, Cuplinskas D, Eisen M, Friend F, Genova Y, Guedon JC, Hagemann M, Harnad S, Johnson R, Kupryte R, La Manna M, Rév I, Segbert M, de Souza S, Suber P, Velterop J Budapest open access initiative: read the Budapest open access initiative [Internet].

[2] Sugimoto CR, Tsou A, Naslund S, Hauser A, Brandon M, Winter D, Behles C, Finlay SC (2014). Beyond gatekeepers of knowledge: scholarly communication practices of academic librarians and archivists at ARL institutions. Coll Res Libr.

[3] ACRL Scholarly Communications Committee (2003). Principles and strategies for the reform of scholarly communication 1 [Internet].

[4] National Institutes of Health (2016). NIH public access policy details [Internet].

[5] Medical Library Association (2016). MLA committees [Internet].

[6] Association of Academic Health Sciences Libraries Membership [Internet].

[7] Cooper ID, Crum JA (2013). New activities and changing roles of health sciences librarians: a systematic review, 1990–2012. J Med Libr Assoc.

[8] Radom R, Feltner-Reichert M, Stringer-Stanback K (2012). Organization of scholarly communication resources. SPEC kit 332.

[9] Malefant KJ (2010). Leading change in the system of scholarly communication: a case study of engaging liaison librarians for outreach to faculty. Coll Res Libr.

[10] Kirchner J (2009). Scholarly communications: planning for the integration of liaison librarian. Res Libr Issues.

