# Supplementary material for: Investigating the need for scholarly communications positions in Association of Academic Health Sciences Libraries member institutions
Source: J Med Libr Assoc. 2017 Apr;105(2):145–9. doi: 10.5195/jmla.2017.208 (PMC5370605; doi:10.5195/jmla.2017.208)
Supplement: Appendix [file jmla_apr17_mears_app.pdf]

JMLA Journal of the Medical Library Association

Supplemental content to
J Med Libr Assoc. Apr;105(2): dx.doi.org/10.5195/jmla.2017.208
www.mlanet.org
© Mears, Bandy 2017

# Investigating the need for scholarly communications positions in Association of Academic Health Sciences Libraries member institutions

Kim M. Mears, MA, MLIS; Sandra L. Bandy, MS, AHIP

## APPENDIX

### Survey

**Scholarly Communications Services in Health Sciences Libraries**

You are being invited to participate in a research project to study scholarly communications in health sciences libraries because you are a director of a health sciences library. The survey asks questions about scholarly communications services offered at your library. It should take you approximately 5 minutes to complete.

The results of this project will be used for a future publication. Through your participation we hope to understand the types of scholarly communications services offered at your library and to identify if there is a trend of hiring librarians designated for these services within health sciences libraries.

There are no known risks to you if you decide to participate in this survey. There is no direct benefit to you for participating in this study. The alternative would be not participating in the study. We will not share any information that identifies you with anyone outside the research group which consists of Sandra Bandy, Chair of Content Management, and Kim Mears, Scholarly Communications Librarian, for the Robert B. Greenblatt, M.D. Library at Georgia Regents University (GRU).

We will keep your information confidential and the survey will not contain information that will personally identify you. All data are stored in a password protected electronic format. The results of this study will be used for scholarly purposes only and may be shared with Georgia Regents University.

I hope you will take the time to complete this questionnaire; however, if you agree to complete the survey you are not required to answer all the questions or complete it. Your participation is voluntary and there is no penalty if you do not participate. If you have any questions or concerns about completing the questionnaire, about being in this study, or to receive a summary of our findings you may contact Sandra Bandy at sbandy@gru.edu and 706-721-0299 or Kim Mears at kmears@gru.edu and 706-721-8789.

If you have any questions or concerns about the "rights of research subjects," you may contact GRU's IRB Office at (706) 721-1483, Project: [702855-2].

Sincerely,

Sandra Bandy, MS, AHIP, sbandy@gru.edu
Kim Mears, MLIS, AHIP, kmears@gru.edu

1

JMLA Journal of the Medical Library Association

SC
Supplemental content to
J Med Libr Assoc. Apr;105(2): dx.doi.org/10.5195/jmla.2017.208
www.mlanet.org
© Mears, Bandy 2017

1. Clicking on the "Agree" button below indicates that:

• you have read the above information
• you voluntarily agree to participate
• you are at least 18 years of age

If you do not wish to participate in the research study, please decline participation by clicking on the "Disagree" button

- ◯ Agree
- ◯ Disagree

2

JMLA Journal of the Medical Library Association

SC
Supplemental content to
J Med Libr Assoc. Apr;105(2): dx.doi.org/10.5195/jmla.2017.208
www.mlanet.org
© Mears, Bandy 2017

## Scholarly Communications Services in Health Sciences Libraries

2. Which scholarly communications services does your library offer? Please check all that apply.

- ☐ Managing an Institutional Repository
- ☐ Assist researchers in complying with the NIH Public Access Policy
- ☐ Education on federal mandates and directives (OSTP Directive, FASTR)
- ☐ Authors' Rights Management
- ☐ Education on Fair Use and Copyright
- ☐ Consultations on publishing (traditional and open access journals)
- ☐ Research Data Management Planning
- ☐ Open access publishing funds
- ☐ Education on researcher identifiers (ORCID, ResearcherID, ScopusID)
- ☐ Support e-science initiatives
- ☐ Assistance in measuring Impact of scholarly works
- ☐ Other (please specify)

3. Does your university have an open access resolution and/or mandate?

- ○ Yes, we have a resolution
- ○ Yes, we have a mandate
- ○ No to both

4. Within the past 2 years, did your library participate in International Open Access Week?

- ○ Yes
- ○ No

5. Does your library have a webpage dedicated to scholarly communications?

- ○ Yes
- ○ No

Supplemental content to
J Med Libr Assoc. Apr;105(2): dx.doi.org/10.5195/jmla.2017.208
www.mlanet.org
© Mears, Bandy 2017

6. Based on your answers to the previous questions in this survey, do you see the need for a full time Scholarly Communications Librarian?

- ◯ Yes
- ◯ No

7. Do you have a librarian assigned to scholarly communications initiatives?

- ◯ Yes
- ◯ No

4

JMLA Journal of the Medical Library Association

Supplemental content to
J Med Libr Assoc. Apr;105(2): dx.doi.org/10.5195/jmla.2017.208
www.mlanet.org
© Mears, Bandy 2017

## Scholarly Communications Services in Health Sciences Libraries

8. What is the librarian's title?

9. Please offer your comments, and if you would like a summary of the results, provide your contact information.
